# Methicillin-Resistant *Staphylococcus aureus* (MRSA) Clonal Replacement in a Malaysian Teaching Hospital: Findings from an Eight-Year Interval Molecular Surveillance

**DOI:** 10.3390/antibiotics10030320

**Published:** 2021-03-19

**Authors:** Mohd Azrul Hisham Ismail, Norhidayah Kamarudin, Muttaqillah Najihan Abdul Samat, Raja Mohd Fadhil Raja Abdul Rahman, Saberi Saimun, Toh Leong Tan, Hui-min Neoh

**Affiliations:** 1 UKM Medical Molecular Biology Institute (UMBI), Universiti Kebangsaan Malaysia, Kuala Lumpur 56000, Malaysia; azrulhisham@ukm.edu.my (M.A.H.I.); ashranfd86@gmail.com (R.M.F.R.A.R.); saberi@ppukm.ukm.edu.my (S.S.); 2 Kulliyyah Of Medicine, International Islamic University Malaysia, Pahang 25200, Malaysia; norhidayahkamarudin@iium.edu.my; 3 Department of Medical Microbiology and Immunology, Faculty of Medicine, Universiti Kebangsaan Malaysia, Kuala Lumpur 56000, Malaysia; muttaqillah@ppukm.ukm.edu.my; 4 Department of Emergency Medicine, Faculty of Medicine, Universiti Kebangsaan Malaysia, Kuala Lumpur 56000, Malaysia; sebastianttl@yahoo.co.uk

**Keywords:** MRSA, molecular surveillance, SCC*mec* typing, toxin typing, *spa* typing, antimicrobial susceptibility

## Abstract

Periodical surveillance on nosocomial pathogens is important for antimicrobial stewardship and infection control. The first methicillin-resistant *Staphylococcus aureus* (MRSA) molecular surveillance in Hospital Canselor Tuanku Muhriz (HCTM), a Malaysian teaching hospital, was performed in 2009. The dominant clone was identified as an MRSA carrying SCC*mec* type III-SCC*mercury* with *ccrC* and *sea*+*cna* toxin genes. In this study, we report the findings of the second HCTM MRSA surveillance carried out in 2017, after an interval of 8 years. Antibiotic susceptibility testing, SCC*mec*, toxin gene, and *spa* typing were performed for 222 MRSA strains isolated in 2017. Most strains were resistant to ciprofloxacin, erythromycin, clindamycin, cefoxitin, and penicillin (*n* = 126, 56.8%), belong to SCC*mec* type IV (*n* = 205, 92.3%), *spa* type t032 (*n* = 160, 72.1%) and harboured *seg*+*sei* toxin genes (*n* = 172, 77.5%). There was significant association between resistance of the aforementioned antibiotics with SCC*mec* type IV (*p* < 0.05), t032 (*p* < 0.001), and *seg*+*sei* carriage (*p* < 0.05). Results from this second MRSA surveillance revealed the occurrence of clonal replacement in HCTM during an interval of not more than 8 years. Investigation of the corresponding phenotype changes in this new dominant MRSA clone is currently on-going.

## 1. Introduction

Methicillin resistant *Staphylococcus aureus* (MRSA) has been listed as “High” in the World Health Organization (WHO)’s priority pathogens list for research and development of new antibiotics [1]. The pathogen is notorious for acquiring resistance to almost all groups of available antibiotics, with various multi-drug resistant lineages isolated from many countries in the world [2]. It also causes a range of illnesses from minor skin infections to life-threatening diseases, via its carriage of toxin genes and pathogenicity islands [3,4].

Due to MRSA’s medical importance, the pathogen is consistently studied in many surveillance initiatives [5,6,7,8,9]. Indeed, surveillance on MRSA antibiotic susceptibility is routinely carried out and reported in Malaysia by the Ministry of Health, in conjunction with Malaysia’s Malaysian National Surveillance on Antimicrobial Resistance (NSAR) and the country’s conformance to WHO’s Global Antimicrobial Resistance Surveillance System (GLASS) initiative [10,11]. In addition, a few Malaysian hospitals have also conducted molecular surveillance on local MRSA strains [12,13,14,15,16]. 

Hospital Canselor Tuanku Muhriz (HCTM) is a teaching hospital located in Cheras, Kuala Lumpur. It is a 900-bed hospital that began operations in 1997. In 2009, the first molecular surveillance on MRSA infections of this hospital was conducted, where a total of 318 MRSA strains (purified from the first isolate of each infection) was included into the study. It was found that most of the MRSA strains in 2009 were of the SCC*mec* type III-SCC*mercury* with *ccrC* genotype, and carried *sea + cna* toxin genes [17]. The majority of the strains were also resistant to ciprofloxacin, erythromycin, and gentamicin. This MRSA (habouring SCC*mec* type III-SCC*mercury* with *ccrC*, *sea*, *cna* and resistant to ciprofloxacin, erythromycin, and gentamicin) also appeared to be circulating in Thailand during the same period, and in Singapore before 2010 (Ang et al., manuscript in preparation).

We recently completed a follow-up surveillance on the MRSA strains isolated from HCTM in the year 2017. This report describes the changes found in MRSA clones of our hospital, compared to the year 2009. 

## 2. Results

### 2.1. Bacterial Strains and Patient Demographic Profiles

A total of 222 MRSA infections in patients from various wards of HCTM were recorded in 2017. Table 1 shows demographic data of patients from whom MRSA was isolated in this surveillance. The majority of the patients from this study were males (64.0%), of the Malay heritage (54.0%), and above 50 years old (69.5%). Most MRSA infections occurred in the medical ward (30.6%) and were isolated from swabs (23.9%).

### 2.2. Antimicrobial Susceptibility Testing

Table 2 shows antibiotic resistance profiles of tested strains. All strains were resistant to cefoxitin and penicillin, with more than half of the MRSA strains also resistant to ciprofloxacin (*n* = 183, 82.4%), erythromycin (*n* = 172, 77.5%) and clindamycin (*n* = 149, 67.1%). We also observed seven (3.2%), three (1.4%), and one (0.5%) of the strains exhibiting resistance to trimethoprim-sulfamethoxazole, doxycycline, and linezolid, respectively. All tested strains were susceptible to vancomycin, with MIC values between 0.1–2.0 mg/L.

### 2.3. SCCmec, Toxin Gene and Spa Typing

The majority (*n* = 205, 92.3%) of tested strains were typed as SCC*mec* type IV. Eight (3.6%) MRSA strains were of type V, and only one (0.5%) strain was SCC*mec* type III-SCC*mercury*. Eight (3.6%) MRSA strains were untypeable. For toxin gene typing, the most prevalent toxin gene profile for our tested strains was *seg+sei* (*n* = 172, 77.5%). Notably, no MRSA harboured the *eta* and *etb* genes. Table 3 shows toxin gene profiles of tested MRSA strains. 

For *spa* typing, 33 *spa* types were identified for our MRSA strains; nevertheless, 25 of these *spa* types were represented by only a single strain. The most prevalent *spa* type was t032 (*n* = 160, 72.1%), followed by t304 (*n* = 17, 7.7%) (Table 4).

### 2.4. Association between Typing Methods, SCCmec Type and Patient Demographic Data

We found a significant association between ciprofloxacin, erythromycin, cefoxitin, penicillin, and clindamycin resistance with SCC*mec* type IV (*p* < 0.05), *spa* type t032 (*p* < 0.001) and carriage of *seg* and *sei* (*p* < 0.05). Accordingly, SCC*mec* IV was associated with *spa* type t032 (*p* < 0.001) and carriage of *seg* and *sei* (*p* < 0.05). *spa* type t032 was associated with carriage of *seg* and *sei* (*p* < 0.001). 

On the other hand, we did not find any association between SCC*mec* type with patient gender (*p* < 0.738), ethnicity (*p* < 0.215), ward of admittance (*p* < 0.524), and source of MRSA isolation (*p* < 0.990). Interestingly, SCC*mec* type IV was associated with older patient age (*p* < 0.014).

## 3. Discussion

This report describes the second molecular surveillance study on MRSA strains isolated from HCTM after an interval of 8 years. The first study was conducted on MRSA strains isolated in 2009 [17]. Parameters of the current surveillance were like those in 2009 with some exceptions. We substituted pulsed-field gel electrophoresis (PFGE) with *spa* typing, due to the phasing out of the technology in our institution, and also only focused on the staphylococcal entero- and exfoliative toxins for toxin gene typing in this current surveillance.

We observed an interesting transition in characteristics of the dominant MRSA of our hospital after an interval of 8 years. The dominant MRSA in HCTM isolated in the year 2009 was reported to be a SCC*mec* type III-SCC*mercury* with *ccrC* clone, harbouring *sea + cna* toxin genes, and resistant to ciprofloxacin, erythromycin, and gentamicin (in addition to penicillin and cefoxitin) [17]. This clone was usually associated with hospital-acquired infections [2]. Interestingly, during an interval of (at the most) 8 years, the dominant MRSA in HCTM has been replaced by a new clone: one that harboured SCC*mec* type IV, the *seg + sei* genes, and with resistance towards ciprofloxacin, erythromycin, clindamycin, penicillin and cefoxitin. Gentamicin resistance was not as prevalent in our current MRSA strains (*n* = 12, 5.4%), as compared to 8 years ago (*n* = 276, 86.8%). We suspect the change in gentamicin resistance and enterotoxin profile is due to MRSA clonal replacement in HCTM. The HCTM Antimicrobial Stewardship (AMS) team was established in 2011, and, subsequently, there was a revised vancomycin trough level (15 mg/L to 20 mg/L) requirement. It remains to be investigated if the aforementioned changes in trough level created additional antibiotic selection pressure on the MRSA strains. Communication with HCTM’s Infection Control Unit revealed no changes in the unit’s policy and standard operating procedure for MRSA carriage control from 2009–2017.

Some of the earliest reports of SCC*mec* type IV MRSA isolates were from the U.S.A. and Australia; these MRSA strains were mostly from community-acquired infections [18,19]. SCC*mec* type IV MRSA strains have smaller SCC*mec* cassettes and have been reported to carry fewer antibiotic resistance genes compared to hospital-acquired MRSA strains clones, which are usually of SCC*mec* types I, II, or III [20]. Nevertheless, SCC*mec* type IV MRSA strains have been reported to be “fitter” and to replicate more efficiently than the larger SCC*mec* type III MRSA strains [2,21]. Notably, we have also observed faster growth and shorter doubling time for our 2017 cohort of MRSA strains, compared to those isolated from 2009 [22]. We are currently investigating if these phenotype changes in conjunction with MRSA clonal replacement affect patient clinical course and mortality. We were only granted ethical approval to obtain basic patient demographic data for both 2009 and 2017 surveillance studies; our current findings of clonal replacement and possible associated phenotype changes will provide us with stronger rationale to seek the university research ethics committee’s approval for a larger, detailed investigation.

Intriguingly, this hospital- to community-acquired genotype shift has been observed in hospitals from different regions of the world [21,23,24,25,26,27]. In fact, clonal replacement of SCC*mec* type III to type IV MRSA strains was reported to have occurred in hospitals of neighbouring Singapore, right after the turn of the century, and the prevalence of SCC*mec* type IV in the republic has since overtaken that of type III [25].

In Malaysia, molecular surveillance studies for MRSA strains, including the first study for HCTM MRSA strains, were mostly initiated after the millennium, where the dominant genotype for MRSA strains isolated during this first phase of surveillance (2003 until 2012) was reported to be SCC*mec* type III-SCC*mercury* with *ccrC* [13,14,17,28]. Most of the MRSA strains from this phase carried the *sea* enterotoxin [15,17,28]; were resistant to ciprofloxacin, erythromycin, clindamycin, gentamicin, penicillin and cefoxitin [14,17]; and were of *spa* type t037 [15,16,28].

Comparatively, there were fewer second phase surveillance studies of Malaysian MRSA strains (for strains isolated after 2012) [12,29]. Nonetheless, like our findings, two other studies of this phase also noted the higher prevalence of SCC*mec* type IV compared to other SCC*mec* types. Only one of these studies (on paediatric patients attending Likas Hospital, Sabah, Borneo) performed *spa* typing on their tested MRSA strains [29]. Interestingly, despite similarity in SCC*mec* type (type IV), distribution of the MRSA *spa* types found in that study was very different to that of ours; t019 was the only *spa* type found in both the Likas study (*n* = 6, 60.0%) and ours (*n* = 2, 0.9%). Of note, Borneo island, where Likas Hospital is situated, is separated from Peninsular Malaysia by the South China Sea, and MRSA molecular surveillance studies from this region remain few. We suspect the MRSA genotypes of Borneo island will be different from those isolated from Peninsular Malaysia. From our experience and observation of results from the first phase molecular surveillance, MRSA strains isolated from hospitals located in Peninsular Malaysia are genotypically similar. Nevertheless, it remains to be investigated if these Peninsular MRSA strains evolutionarily diverged according to sampling sites, or remain similar after the first phase surveillance [13,14,17,28]. We did not perform *spa* typing during the first HCTM MRSA surveillance. For future surveillance, we intend to continue utilizing the more discriminatory *spa* typing for strain characterization.

So far, no MRSA isolate in Malaysia has been reported to be fully resistant to vancomycin, though incidence of hetero-VISAs (vancomycin intermediate *S. aureus*) and vancomycin MIC creep have been reported [10,13,30,31]. We did not perform vancomycin MIC determination for our MRSA strains during the first phase of HCTM’s surveillance. Nevertheless, for this current surveillance, with vancomycin MIC determination, we found that 3.2% of our tested MRSA strains had vancomycin MICs of ≥1.5 mg/L; this prevalence shall be used as a benchmark to determine the occurrence of MIC creep in our subsequent surveillance studies. We also included the susceptibility testing for three antibiotics, namely trimethoprim-sulfamethoxazole, doxycycline, and linezolid, which were newly introduced into HCTM antibiotic formulary in this current surveillance and will be monitoring our MRSA strains’ susceptibilities towards these antibiotics in future surveillance.

## 4. Materials and Methods

### 4.1. Bacterial Strains and Patient Demographic Profiles

This follow-up surveillance was carried out in all wards of HCTM in 2017. The first isolate of each MRSA infection was collected, colony-purified, and stocked as strains with 40% glycerol at −80 °C until use. Isolates were confirmed to be MRSA via cefoxitin (30 µg) resistance using disc diffusion susceptibility testing (Oxoid Microbiology Products, Thermo Fisher Scientific, Hampshire, United Kingdom) [32]. Research approval for this study was obtained from the Research Ethics Committee, Universiti Kebangsaan Malaysia (UKM PPI/111/8/JEP-2016-419). Corresponding patient demographic data (gender, ethnicity, age, ward of admittance and source of MRSA isolation) of each tested strain was also recorded.

### 4.2. Antimicrobial Susceptibility Testing

Antimicrobial susceptibilities of all MRSA strains were tested via disc diffusion using Mueller-Hinton agar (Becton, Dickinson and Company, Franklin Lakes, NJ, USA) (CLSI, 2007). Tested antibiotics and their concentrations were ciprofloxacin (5 µg), erythromycin (15 µg), fusidic acid (10 µg), gentamicin (10 µg), cefoxitin (1 µg), penicillin (10 U), chloramphenicol (30 µg), clindamycin (2 µg), mupirocin (5 µg), teicoplanin (30 µg), rifampicin (5 µg), trimethoprim-sulfamethoxazole (25 µg), doxycycline (5 µg), linezolid (30 µg) and vancomycin (30 µg) (Oxoid Microbiology Products, Thermo Fisher Scientific, Hampshire, United Kingdom). Vancomycin susceptibility was also tested using E-test antibiotic strips (bioMérieux SA, Marcy l’Etoile, France). Susceptibility results for tested antibiotics were categorized as “susceptible” or “resistant” according to Clinical & Laboratory Standards Institute (CLSI) guidelines [32]; vancomycin minimum inhibitory concentrations (MIC) for tested strains were recorded.

### 4.3. SCCmec, Toxin Gene and Spa Typing

Chromosomal DNA from tested strains was extracted using a Qiagen DNeasy Blood and Tissue extraction kit (Qiagen Inc., Maryland, United States) according to manufacturer instructions. Multiplex PCR was used to determine the strains’ SCC*mec* type [17,33]. All strains were typed via PCR assays for enterotoxins A (*sea*), B (*seb*), C (*sec*), D (*sed*), E (*see*), G (*seg*), I (*sei*), and exfoliative toxins (*eta* and *etb*) [17,34]. For *spa* typing, the polymorphic X region of the protein A gene (*spa*) was amplified by PCR and sequenced [35]. *spa* types were then determined with the Ridom StaphType software (Ridom GmbH, Münster, Germany).

### 4.4. Association between Typing Methods, SCCmec Type and Patient Demographic Data

Statistical analysis was done using IBM SPSS Statistics version 21 (IBM). Association between antibiotic resistance and molecular typing (SCC*mec*, *spa* and toxin gene), as well as between SCC*mec* type and patient demographic data, were determined using Fisher’s exact test, where *p* < 0.05 was considered as statistically significant.

## 5. Conclusions

We completed the second molecular surveillance for MRSA strains isolated in HCTM. During an interval of no more than 8 years, clonal replacement of MRSA strains in our hospital has occurred. We are currently carrying out investigations to determine the effects of this clonal replacement towards patient clinical outcome. With continuous decrease in sequencing costs, we aim to continue including the more discriminatory *spa* typing for future studies, as well as to select representative strains for whole-genome surveillance.

## Figures and Tables

**Table 1:** Demographic data of patients (*n* = 222) from whom MRSA was isolated in this study.

Demographic Factor	*n* (%)
**Gender**	
Male	142 (64.0)
Female	80 (36.0)
**Ethnicity**	
Malay	122 (55.0)
Chinese	80 (36.0)
Indian	16 (7.2)
Others	4 (1.8)
**Age Group**	
0–10	12 (5.4)
11–20	8 (3.6)
21–30	8 (3.6)
31–40	19 (8.6)
41–50	21 (9.5)
51–60	47 (21.2)
61–70	51 (23.0)
71–80	41 (18.5)
81–90	12 (5.4)
91–100	3 (1.4)
**Ward of Admittance**	
Intensive care unit	15 (6.8)
Emergency	22 (9.9)
Ear, Nose Throat	1 (0.5)
Day-Care Ward for HCTM Staff	1 (0.5)
Medical	68 (30.6)
Obstetrics & Gynaecology	6 (2.7)
Oncology	7 (3.2)
Orthopaedic	52 (23.4)
Otorhinolaryngology	1 (0.5)
Paediatric	8 (3.6)
Surgical	38 (17.1)
Designated Ward for HCTM Staff	3 (1.4)
**Source of MRSA Isolation**	
Bronchoalveolar lavage	3 (1.4)
Blood	43 (19.4)
Body fluid	1 (0.5)
Bone	3 (1.4)
Nasal swab	1 (0.5)
Nasopharyngeal aspirate	1 (0.5)
Pus	12 (5.4)
Sputum	41 (18.5)
Wound swab	53 (23.9)
Tissue	44 (19.8)
Tracheal aspirate	17 (7.7)
Urine	3 (1.4)

**Table 2:** Antibiotic resistance of tested MRSA isolated from HCTM in 2017.

Antibiotic Resistance	*n* (%)
Resistance Profiles *	*n* (%)
cip + ery + fus + fox + pen + clin+ tei + sxt + lzd	1 (0.5)
cip + ery + gen + fus + fox + pen + clin + mup + sxt + dox	1 (0.5)
cip + ery + gen + fus + fox + pen + clin + rif + sxt	1 (0.5)
cip + ery + gen + fus + fox + pen + clin + rif	1 (0.5)
cip + ery + fox + pen + clin + mup	1 (0.5)
cip + ery + fus + fox + pen + clin	5 (2.3)
cip + ery + gen + fus + fox + pen	1 (0.5)
cip + ery + gen + fox + pen	1 (0.5)
cip + ery + fox + pen	15 (6.8)
cip + fox + pen	20 (9.0)
cip + ery + fox + pen + clin	126 (56.8)
cip + ery + fox + pen + clin + rif	1 (0.5)
cip + ery + fox + pen + mup	1 (0.5)
cip + ery + fox + pen + clin + mup + sxt	1 (0.5)
cip + ery + gen + fox + pen + sxt	1 (0.5)
cip + ery + gen + fox + pen + clin + dox	1 (0.5)
cip + gen + fus + fox + pen	1 (0.5)
cip + fox + pen + sxt	2 (0.9)
cip + fox + pen + clin	1 (0.5)
cip + gen + fox + pen	1 (0.5)
ery + gen + fox + pen + clin + rif	1 (0.5)
ery + fox + pen + clin	7 (3.2)
ery + fox + pen + clin + dox	1 (0.5)
ery + fox + pen	5 (2.3)
gen + fox + pen	1 (0.5)
gen + fus + fox + pen	1 (0.5)
fox + pen	23 (10.4)
**Antibiotic**	***n* (%)**
ciprofloxacin	183 (82.4)
erythromycin	172 (77.5)
gentamicin	12 (5.4)
fusidic acid	12 (5.4)
cefoxitin	222 (100)
penicillin	222 (100)
clindamycin	149 (67.1)
mupirocin	4 (1.8)
teicoplanin	1 (0.5)
rifampicin	4 (1.8)
trimethoprim-sulphamethoxazole	7 (3.2)
doxycycline	3 (1.4)
linezolid	1 (0.5)

* cip, ciprofloxacin; ery, erythromycin; gen, gentamicin; fus, fusidic acid; fox, cefoxitin, pen, penicillin; chlo, chloramphenicol; clin, clindamycin; mup, mupirocin; rif, rifampicin; sxt, trimethoprim- sulfamethoxazole; dox, doxycycline; lzd, linezolid.

**Table 3:** Toxin gene profiles of MRSA strains isolated in 2017 from HCTM.

Toxin Gene Profile	*n* (%)
*sea*	19 (8.6)
*sea + seb*	1 (0.5)
*sea + seg + sei*	1 (0.5)
*seb*	1 (0.5)
*sec*	1 (0.5)
*sec + seg + sei*	2 (0.9)
*seg*	11 (5.0)
*seg + sei*	172 (77.5)
*sei*	4 (1.8)
toxin gene not detected	10 (4.5)

**Table 4:** *spa* typing of MRSA strains isolated in 2017 from HCTM.

*Spa* Type	*n* (%)
t002	3 (1.4)
t005	1 (0.5)
t019	2 (0.9)
t020	1 (0.5)
t021	1 (0.5)
t022	3 (1.4)
t025	1 (0.5)
t032	160 (72.1)
t034	1 (0.5)
t037	2 (0.9)
t10159	2 (0.9)
t1081	1 (0.5)
t1198	1 (0.5)
t127	1 (0.5)
t1302	1 (0.5)
t1332	1 (0.5)
t1379	1 (0.5)
t1476	1 (0.5)
t15595	1 (0.5)
t18189	2 (0.9)
t18190	1 (0.5)
t18516	1 (0.5)
t2236	1 (0.5)
t304	17 (7.7)
t315	1 (0.5)
t379	1 (0.5)
t3841	1 (0.5)
t3887	1 (0.5)
t4184	1 (0.5)
t437	1 (0.5)
t513	1 (0.5)
t631	1 (0.5)
t904	1 (0.5)
non-typeable	6 (2.7)

## Data Availability

The data presented in this study are available in article.
